# Perceived Learning Needs of Hospitalised Cancer Patients and Nurses

**DOI:** 10.1002/nop2.70163

**Published:** 2025-02-14

**Authors:** Xi Zhang, Meiliyang Wu, Ye Chen, Yisui Su, Tieying Zeng

**Affiliations:** ^1^ Department of Nursing, Tongji Hospital, Tongji Medical College Huazhong University of Science and Technology Wuhan China; ^2^ School of Nursing, Tongji Medical College Huazhong University of Science and Technology Wuhan China

**Keywords:** cancer patients, health education, learning needs, nurses, perception

## Abstract

**Aim:**

To understand the differences in perceived learning needs between cancer patients and nurses.

**Design:**

A cross‐sectional descriptive design.

**Methods:**

Data were collected from 1147 cancer patients and 1000 nurses using a convenience sampling method from February 2022 to June 2023. Sociodemographic data of patients and nurses were collected through a self‐designed general information questionnaire. Patients and nurses completed the Revised Chinese version of the Patient Learning Needs Scale to identify their perceptions of the learning needs and the difference between patient and nurse knowledge of needs.

**Results:**

According to cancer patients and nurses, the top learning needs were information on complications and symptoms. In contrast, patients perceived the lowest priority learning needs to be disease‐related issues, while nurses perceived the lowest priority learning needs to be daily treatment and activities.

**Conclusions:**

Patients and nurses have different views on the essential contents to be learned, highlighting the importance of considering these two aspects in formulating health education programmes.

**Patient or Public Contribution:**

Participating cancer patients and nurses took the time to complete the questionnaire during the data collection phase and answered the questions sincerely.

## 1 Introduction

At present, cancer is the leading cause of death worldwide. The World Health Organisation counted nearly 10 million deaths (or nearly one in six) caused by cancer in 2020 (Deo et al. 2022). China's cause of death surveillance data set for 2021 shows that cancer is the second cause of death, accounting for 23.23% of the global total deaths (Chinese Center for Disease Control and Prevention 2021). It is estimated that by 2040, the number of deaths due to cancer in China will reach 5.07 million (Cao et al. 2021).

Having cancer is a major traumatic event, and patients face many difficulties and challenges, including the severe impact of the disease on their physical health, multiple emotional disorders, adverse reactions to daily life and so on (Emery et al. 2022; Kan et al. 2023). Of all the medical staff, the contact between the nurses and the patients is the most direct, and the nurses play a vital role in the care of the patients (Ajibade 2021). Health education is one of the basic duties of nurses, which includes providing patients with relevant health knowledge and guidance, helping them to understand and grasp their health conditions and taking appropriate preventive and curative measures (Du et al. 2015; Gorod et al. 2021). The literature suggested that educational and supportive interventions provided by nurses positively impact the quality of life (QOL) of cancer patients (Sajjad et al. 2016). Personalised health education tailored to patients' learning needs can help patients better overcome the challenges of their illness, improve adherence and satisfaction with treatment and reduce the incidence of unplanned hospital readmissions (Deniz et al. 2017; Pueyo‐Garrigues et al. 2019).

Previous researches have confirmed that cancer patients have a series of learning needs in the psychological, physiological and life fields at their disease stage (Aunan et al. 2021; O'Dea et al. 2022). Wu et al. (2022) found that most cancer patients have diverse and complex learning needs, which may relate to several areas, such as disease knowledge, side effect management, lifestyle changes, psychological support and rehabilitation. Patients need to be equipped with different kinds of information to better cope with their disease and treatment. However, many patients reported that their health education was unrelated to their perceived learning needs (Cheng et al. 2016). Nurses often tend to make wrong judgements about the learning needs of patients and provide information that patients do not need (Suhonen et al. 2005). In addition, nurses in oncology care more about information related to the disease process than important information related to patients' lives (Labbe‐Pinlon et al. 2023; Wu et al. 2022).

For hospitalised cancer patients, it is very important to understand the disease development process, treatment procedures and possible outcomes (Germain et al. 2017). Cancer patients might undergo different treatment modalities, which include chemotherapy, surgery, radiotherapy and so on. Patients' learning needs in different situations varied (Soper 2020). For example, chemotherapy patients might experience side effects, which can affect their self‐efficacy against disease, affecting their quality of life and reducing confidence about fighting disease (Compaci et al. 2011). A good nursing relationship should be a process of interaction between nurses and patients in which each party can perceive the needs of the other in a timely manner, determine goals through communication, explore ways to achieve goals and ultimately achieve goals (Düdener and Hallaç 2023; Kottschade et al. 2014). Therefore, nurses should timely understand how patients feel about their learning needs and compare their feelings about the learning needs of hospitalised cancer patients with those of cancer patients to identify problems and then analyse the reasons, which is conducive to carrying out high‐quality patient health education in the future.

In recent years, in order to improve the utilisation of healthcare resources, the length of hospital stay for patients has been significantly reduced (Lam et al. 2016). With the shortening of hospitalisation time, role adaptation and self‐care of cancer patients returning home from the hospital are particularly important (Mistiaen et al. 2007). Health education of hospitalised patients is limited by time, knowledge level and learning ability of patients, and it is impossible to carry out comprehensive education in a limited time. At this time, we should consider which needs are most urgent and most beneficial to preventing and treating their diseases. However, the existing health education mainly starts from the role of nursing staff and needs a deeper understanding of the learning needs of patients (Cheng et al. 2016; Zheng et al. 2014).

In view of the above, this study aimed to investigate the learning needs of both nurses and cancer patients. Most importantly, this study intended to elucidate whether there are differences in perceived learning needs between cancer patients and nurses.

## 2 Methods

### 2.1 Study Design

A cross‐sectional descriptive design was used in this study. The survey data were collected from the oncology departments of general hospitals in the central region of China from February 2022 to June 2023. The oncology departments of these general hospitals receive patients from all over the country.

### 2.2 Samples

The study recruited samples using a convenient sampling method. Inclusion criteria for hospitalised cancer patients were as follows: (1) age ≥ 18 years; (2) relatively stable physiological status and knowledge of their cancer diagnosis and (3) informed consent and voluntary participation in the study. Exclusion criteria were as follows: (1) suffering from mental illness; (2) unable to complete the questionnaire independently. This study included 1200 eligible patients who completed the questionnaire. Deleting data where the time to complete the questionnaire is < 5 min or the data is incomplete 20%. A total of 1147 valid questionnaires were analysed, with a validity rate of 95.58%.

Nurses caring for these hospitalised cancer patients were also invited to participate. The inclusion criteria for nurses were as follows: (1) obtaining a nurse's licence and being a registered nurse; (2) working in the care of cancer patients ≥ 6 months and (3) volunteering to participate in the study. The study included 1100 eligible nurses, of which 52 refused to participate because they were too busy at work, and 1048 nurses completed the questionnaire, deleting the data where the time taken to complete the questionnaire was < 5 min. A total of 1000 valid questionnaires were analysed, with a validity rate of 95.42%.

### 2.3 Data Collection

Questionnaires were distributed and collected using Wenjuanxing software (https://www.wjx.cn/) in the oncology departments of eight general hospitals. The purpose and significance of the study were introduced to cancer patients and nurses before questionnaire distribution, and the confidentiality of the information was explained. The questionnaires were filled out anonymously to obtain the cancer patients' and nurses' support and cooperation, and informed consent was signed. Each participant completed the survey in an average of 10–15 min.

### 2.4 Measurements

Based on the review of relevant literature (Germain et al. 2017; Kim et al. 2013; Ringash 2017), the researchers self‐designed the general data questionnaire to obtain the general information of the participants. The general information for hospitalised cancer patients included age, gender, educational level, finances and clinical history (e.g., type of cancer, clinical stage, history of treatment and medication). The general information section for nurses included information on gender, education, work experience and so on.

The Patient Learning Needs Scale (PLNS) was developed by Bubela et al. (1990) and translated into Chinese by Yao and Chen (2009) to measure the learning needs as perceived by patients themselves. The scale consists of five dimensions: community support and care, medication, daily treatment and activities, complications and symptoms and disease‐related issues, with 40 items. This scale uses a Likert 5‐level scoring method: ‘not important’ is 1 point, ‘vitally important’ is 5 points and the score range is 40–200 points. Higher scores indicate that patients have a higher perceived learning need to learn. The Cronbach's α of the Chinese PLNS in this study was 0.98, and the Cronbach's α for the subscales ranged from 0.89 to 0.95.

### 2.5 Ethical Considerations

The study complied with the requirements of the Declaration of Helsinki and was approved by the hospital ethics committee. All participants were fully informed about the purpose and content of the study and provided written informed consent. Participants had the right to withdraw from the study at any time. In case of any psychological discomfort during the study, participants were provided with psychological counselling services at no cost. All participants' information was kept strictly confidential and used only for the purpose of this study.

### 2.6 Data Analysis

Data were analysed using the Statistical Package for the Social Sciences (SPSS) version 24. Descriptive statistics were used to describe the sample. Mean (standard deviation) and frequency (percentage) were calculated to describe the study sample (patients and nurses). Independent *t*‐tests were used to determine differences between patients and nurses on the PLNS scale. A two‐tailed *p* < 0.05 indicated the level of significance.

## 3 Results

### 3.1 Sample Characteristics

A total of 1147 patients with valid questionnaires were included in the data analysis. Patients ranged from 18 to 83 years, with a mean age of 58 (SD = 12.5). Among the patients, 49.8% were male, 50.2% were female, 85.3% were married, 68% had less than a secondary school education and the length of hospital stay (days) was mainly more than 29 days (75.9%, *n* = 871). Detailed patient characteristics are shown in Table 1.

**TABLE 1:** General demographics of hospitalised cancer patients (*n* = 1147).

Variables	*n*	%
Gender		
Male	571	49.8
Female	576	50.2
Age		
18–44 years	125	10.9
45–59 years	448	39.1
60–85 years	574	50.0
Marital status		
Married	978	85.3
Other status	169	14.7
Educational level		
Less than secondary level	780	68.0
Secondary school	309	26.9
College or higher	58	5.1
Finance		
< ¥2000/month	436	38.0
¥2000–3999/month	378	33.0
¥4000–5999/month	240	21.0
> ¥6000/month	93	8.0
Type of cancer		
Lung	435	37.9
Liver	287	25.0
Gynaecological	264	23.0
Stomach	161	14.1
Clinical stage		
Early	413	36.0
Middle	516	45.0
Advanced	137	12.0
Unknow	81	7.0
Therapy methods^a^		
One	281	24.5
Two	563	49.1
More than two	303	26.4
Hospitalisation times (days)		
< 15	126	11.0
15–28	150	13.1
≥ 29	871	75.9

^a^ Patients might have more than one type of therapy methods.

A total of 1000 nurses returned valid questionnaires. Most nurses were female (89%, *n* = 890). Their main nurse was < 30 years old, and their nursing experience was < 5 years for 42% and 6–10 years for 39%. Most nurses had a junior college (41%, *n* = 410) or bachelor's degree (48%, *n* = 480).

### 3.2 Perceptions of the Priority of Learning Needs

Patients believed that several types of information about themselves were important during recovery. Five of the PLNS subscales scored around 2–3. The complications and symptoms subscale was rated as the most important (*M* = 3.96, SD = 1.14), followed by medication, daily treatment and activities and community support care. Patients' lowest score for learning needs was for disease‐related issues (*M* = 2.86, SD = 0.81). The specific contents are shown in Table 2.

**TABLE 2:** Perceptions of learning needs of cancer patients and nurses.

Learning needs	Patient		Nurses	
	Priority rank	Mean (SD)	Priority rank	Mean (SD)
Complications and symptoms	1	3.96 (1.14)	1	4.36 (0.32)
Medication	2	3.69 (1.31)	3	3.98 (0.58)
Daily treatment and activities	3	3.59 (1.17)	5	3.95 (0.41)
Community support care	4	3.36 (1.07)	4	3.96 (0.42)
Disease‐related issues	5	2.86 (0.81)	2	4.01 (0.43)
Total PLNS	3.52 (1.05)		4.05 (0.36)	

Nurses rated three categories of learning needs as moderately important for patients and the remaining two categories as very important. Complications and symptoms were also given the highest priority. In contrast, learning about daily treatment was given the lowest priority.

### 3.3 Comparing Learning Needs Between Cancer Patients and Nurses

When comparing nurses' perceptions of the learning needs of cancer patients with patients' views, there were some differences (see Table 3). Patients reported a lower need for information than did nurses. There was agreement between nurses and patients regarding complications and symptoms as the top‐priority learning need (mean difference 0.40, 95% CI −0.23 to 0.12, *p* = 0.383). However, nurses were more concerned than patients about medication (3.98 vs. 3.69, *p* = 0.001), daily treatment and activities (3.95 vs. 3.59, *p* = 0.002) and disease‐related issues (4.01 vs. 2.86, *p* = 0.003).

**TABLE 3:** Comparison of learning needs between cancer patients and nurses.

Learning needs	Mean difference	95% CI of the difference	*p* ^a^
Complications and symptoms	−0.40	−0.23 to 0.12	0.383
Medication	−0.29	0.02 to 0.24	0.001
Daily treatment and activities	−0.36	0.06 to 0.12	0.002
Community support care	−0.60	−0.16 to 0.10	0.045
Disease‐related issues	−1.15	0.07 to 0.34	0.003
Total PLNS	−0.53	0.04 to 0.28	0.001

Abbreviation: PLNS, Patient Learning Need Scale.

^a^ Independent *t*‐test.

## 4 Discussion

Understanding the patient's perceived learning needs is a core element of any discharge plan (Mosleh et al. 2017). Patients need to acquire the knowledge and skills to self‐manage their illness or recovery process when they return to their home environment (Friesen‐Storms et al. 2015). This study highlighted the most important learning needs identified by hospitalised cancer patients and nurses. Therefore, our findings could serve as a basis for developing effective discharge plans. The results showed that complications and symptoms were considered the top priority for learning needs. In contrast, patients perceived learning needs related to disease‐related issues as less important, while nurses perceived learning needs related to daily treatment and activities as less important. It was noted that nurses and patients disagreed on three learning needs categories: medication, daily treatment and activities and disease‐related problems. These findings suggest that health education for patients during hospitalisation should be based on a realistic assessment of patients' learning needs.

Cancer patients are under tremendous physical and psychological stress, and they are more concerned about specific symptoms such as pain, vomiting, loss of appetite, anxiety, depression and so on (Almasri and McDonald 2023). Therefore, it is no surprise that information about complications and symptoms is a top priority for patients and nurses. This result is consistent with the results of a previous study (Mosleh et al. 2017). Similarly, Papadakos et al. (2012) found that the most important information needed by cancer patients was information about upcoming treatments, side effects of treatments and possible complications and symptoms. Patients need information to recognise and avoid complications, as all of these are unknown to them, in order to better self‐care at home and expect the best possible outcome and prolonged life cycle.

Although both patients and nurses perceived information about medication to be important, patients ranked needs in this area higher than nurses' perceptions of needs in this area. This is consistent with previous studies (Alkubati et al. 2013). One explanation for this might be that medication is an important treatment for cancer patients, and most cancer patients experience a combined approach to medication (Posadas et al. 2017). There is a high demand for learning about the principles of medication, commonly used medications, and treatments. The side effects of medication are another concern for patients. While medication can effectively kill the tumour cells, it has certain damage to the normal cells and has certain negative effects, such as nausea, vomiting, hair loss, mouth ulcers and so on (Kean et al. 2016). As a result, patients might be more dependent on medications to control their health.

The physical, social, psychological and daily activities of cancer patients are all affected differently by the disease (Akyüz et al. 2008). In this study, patients ranked daily treatment and activities as the third highest priority, and nurses ranked it as the lowest. Supporting this finding, we found that learning about daily treatment and activities was one of the concepts that led to changes in an individual's social life (Akkuzu et al. 2018). Nursing theorist Nancy Roper believed that activities of daily living (ADLs) were a concept that formed the basis of personalised care and health maintenance for patients (Albayrak 2013). The study's results showed that patients' learning needs for daily treatment and activities were higher than those perceived by nurses, which is inconsistent with Mosleh et al. (2016) findings. In the specific nursing process, nurses might pay more attention to the patient's physical condition, medication management and observation of the condition, while guidance on activities of daily living might be provided by professionals such as rehabilitators or social workers (Bark et al. 2023; Darawad et al. 2016). Thus, nurses can work with other professionals to provide comprehensive patient care.

Both cancer patients and nurses ranked community support care as the fourth. This finding might indicate that patients and nurses did not recognise the importance of community support nursing in the overall learning needs. Community supportive care plays an important role in the overall care of cancer patients. It can provide integrated support and resources to help patients and families cope with the disease's various challenges (Beesley et al. 2016). However, community supportive care may be underdeveloped as it is a relatively new field, and there may be a lack of relevant resources and professionals in certain regions or healthcare settings (Akkuzu et al. 2018). Another learning need is disease‐related issues, such as the problem of urinary problems in cancer patients, how to deal with family or friends about disease, recovery time and so on. In the present study, the patients considered the learning needs of the disease‐related issues to be the least important, but nurses rated it the second. There is also some bias in the perception of this learning need. Further research is needed to confirm this finding.

Additionally, we found that, as a whole, cancer patients' learning needs scores were lower than those perceived by nurses. Cancer itself is a serious disease, and patients may simultaneously face challenges in physical condition, discomfort during treatment and psychological stress (Driehuis et al. 2023). These factors may reduce the patient's interest and ability to learn. At the same time, patients often need to receive complex information about their treatment, which is often difficult for lay people to understand. Patients may feel confused and unable to assess their learning needs accurately (Cheng et al. 2016; Mosleh et al. 2017). Giving patients information they need most about their learning needs has been shown to have a positive impact on treatment and the overall care process (Kean et al. 2016). In summary, health education for cancer patients about diagnosis, treatment and care requires nurses to assess patients' learning needs accurately.

### 4.1 Limitations

This study has some limitations. First, a convenience sampling method was used to select patients from the oncology departments of eight general hospitals in central China. Although the sample size is sufficient, the sample selection still has some limitations. Future studies could investigate in different countries, regions and hospitals of varying levels to further improve related studies. In addition, this study was a cross‐sectional survey and did not address the different learning needs of nurses and patients, so targeted interventions could be developed in the future.

## 5 Conclusions

Cancer patients and nurses expressed a strong desire to know all the learning needs categories of the PLNS scale, as they rated the content of all the subscales as effective for disease management and recovery. This highlights the urgent need to understand cancer patients' real learning needs and develop comprehensive health education programmes that meet their needs.

Although cancer patients and nurses had different perceptions of learning needs, they had similar views and rated all categories as important or very important. In addition, both cancer patients and their nurses indicated that the most important learning needs were topics related to symptoms and complications, and less attention was given to community support care. This study provides information about cancer patients' learning needs and nurses' perceived learning needs. The learning needs of people with cancer were identified. It provides a theoretical basis for health education of the medical care group. It can provide more appropriate, relevant knowledge to cancer patients, which is important in understanding and recovering from the disease.

## Author Contributions

Xi Zhang, Meiliyang Wu and Tieying Zeng conceived and designed the study. Xi Zhang, Meiliyang Wu, Ye Chen and Yisui Su acquired the data. Xi Zhang, Meiliyang Wu and Tieying Zeng analysed and interpreted the data. Xi Zhang, Meiliyang Wu and Tieying Zeng interpreted the data and critically revised the manuscript. Tieying Zeng supervised the study.

## Conflicts of Interest

The authors declare no conflicts of interest.

## Data Availability

The data that support the findings of this study are available from the corresponding author upon reasonable request. The data are not publicly available due to privacy or ethical restrictions.
